# Structural and Evolutionary Analysis of Non-specific Lipid Transfer Proteins (nsLTPs) in *Cereus* (Cactaceae)

**DOI:** 10.1007/s00239-026-10351-2

**Published:** 2026-10-09

**Authors:** M. I. O. Cardoso, J. A. Teodoro, T. A. Costa-Silva, D. T. Amaral

**Affiliations:** Centro de Ciências Naturais e Humanas, Universidade Federal do ABC (UFABC), Avenida dos Estados, 5001, bloco A, Lab. 504-3, Santo André, São Paulo, 09210-580 Brazil

**Keywords:** *Cereus*, Gene duplication, Lipid transfer proteins, Protein structure, Stress response

## Abstract

The genus *Cereus* (Cactaceae), widely distributed across arid and semi-arid environments of South America, exhibits adaptive capacity that is partly associated with proteins involved in lipid metabolism and stress responses, such as non-specific lipid transfer proteins (nsLTPs). Although well-characterized in model plants, the structural diversity and functional roles of these proteins in cacti remain largely unexplored. In this study, we characterized the nsLTP repertoire of *Cereus* by integrating transcriptomic data, phylogenetic analyses, structural modeling, and ligand-binding site mapping. Structural comparisons revealed conservation of the typical nsLTP fold, with variations mainly localized to loop regions surrounding the binding cavity, suggesting functional plasticity without compromising scaffold stability. Phylogenetic analyses revealed frequent duplication and loss events, as well as species-specific paralogs. Several lineage-specific copies exhibited distinctive loop architectures and pocket geometries relative to their closest orthologs, suggesting rapid structural diversification following duplication. This pattern is consistent with functional fine-tuning after gene duplication. Positive selection analyses identified nine codons with significant evidence of episodic diversifying selection (p < 0.05), some of which mapped near predicted functional regions, supporting the hypothesis that selective pressures have shaped key interaction interfaces. Together, these results provide the first integrated characterization of nsLTPs in *Cereus*, supporting a gate-modulation hypothesis in which structural variation concentrated at tunnel entrances may contribute to functional diversification while preserving the conserved nsLTP fold (four-helix scaffold). Rather than asserting adaptation directly, our findings provide a structural basis and testable predictions for how nsLTP diversification may influence lipid trafficking processes relevant to cuticle/membrane function under abiotic stress.

**Supplementary Information:**

The online version contains supplementary material available at https://doi.org/10.1007/s00239-026-10351-2.

## Introduction

The genus *Cereus* Mill. (Cactaceae, tribe Cereeae) represents a diverse and widely distributed group within the cactus family (Franco et al. 2017). Comprising approximately 31 recognized species, *Cereus* occurs across a variety of South American biomes, ranging from coastal regions to Andean habitats above 3,000 m in altitude (Hunt et al. 2006). This broad distribution reflects a remarkable adaptive capacity to contrasting environmental conditions, including drought and temperature fluctuations typical of xeric and semi-arid ecosystems (Amaral et al. 2021; 2025a). The phylogenetic framework proposed for *Cereus* was later refined by recent phylogenetic analyses employing nuclear orthologous genes and a coalescent-based approach (Bombonato et al. 2020; Taylor et al. 2023). These studies support the genus as monophyletic and, although previous analyses structured it into major clades (Bombonato et al. 2020), the new phylogeny, which tested the preexisting subgeneric classification from 1992, partially recovered three of the four subgenera.

The adaptability of *Cereus* to xeric and semi-arid ecosystems, an adaptation observed at anatomical, physiological, and biochemical levels, is reflected in the production of a wealth of secondary metabolites. In traditional medicine, the cladode, root, and seeds of *Cereus jamacaru* D.C. are used to treat various ailments, including urinary tract infections, kidney inflammation, syphilis, gastritis, and cardiovascular and respiratory disorders (Andrade et al. 2006; Lucena et al. 2013; Palheta et al. 2017; da Silva et al. 2019; Rodrigues Almeida and Gonzaga Fernandez 2025; Teodoro et al. 2025; Cardoso et al. 2026). The phytochemical composition of *C. jamacaru* cladodes reveals the presence of phenethylamine alkaloids, such as hordenine, tyramine, and N-methyltyramine, as well as tyrosine. Notably, tyramine exhibits sympathomimetic activity and a probable cardiotonic effect. Other bioactive compounds identified include flavonoids and tannins, which confer crucial therapeutic properties, including anti-inflammatory, antifungal, antioxidant, and wound-healing activities (Rodrigues Almeida and Gonzaga Fernandez 2025).

Moreover, exploring *Cereus* at the molecular level, particularly through the analysis of its proteins, may uncover key mechanisms underlying the evolutionary history and adaptive radiation of the genus. Among these proteins, non-specific lipid transfer proteins (nsLTPs) are of particular interest due to their structural diversity and functional versatility across plant lineages (Missaoui et al. 2022). These are small (around 9 kDa) and basic proteins broadly distributed across higher plants, typically composed of four to five *α *-helices stabilized by a conserved motif of eight cysteine residues forming four disulfide bonds (D’Agostino et al. 2019). This compact fold creates a hydrophobic tunnel-like cavity that allows nsLTP to bind and transport a wide range of lipid molecules; structural features that not only stabilize under stress conditions but also enable participation in multiple biological roles, including membrane remodelling, cell wall organization, and signaling pathways (Egger et al. 2010; Missaoui et al. 2022).

Previous studies have shown that nsLTPs contribute to plant responses to drought, salinity, cold, oxidative stress, and pathogen attack. In drought-tolerant and xerophytic systems, these proteins have been associated with cuticular lipid deposition, wax accumulation, membrane remodeling, and maintenance of cellular integrity under water deficit conditions. In cacti, *Opuntia streptacantha* nsLTPs have been linked to abiotic stress adaptation and lipid metabolism, suggesting that diversification of this protein family may be relevant to the physiology of drought-adapted lineages (Rojas et al. 2019). Similarly, nsLTPs from other stress-tolerant plants have been associated with improved tolerance to osmotic and drought stress and remodeling of lipid profiles (Xu et al. 2018; Xiao et al. 2023). Despite these advances, nsLTPs remain poorly characterized in non-model cactus lineages. In particular, no integrated structural and evolutionary analysis has been performed for nsLTPs in *Cereus*, a genus that occupies diverse xeric and semi-arid environments in South America. This gap limits our understanding of whether the conserved nsLTP scaffold is accompanied by lineage-specific sequence and structural diversification that could generate testable hypotheses about lipid-binding variation in these plants.

Although recent phylogenomic studies have refined the evolutionary framework of *Cereus*, the evolutionary diversification and structural variation of gene families potentially associated with stress-related processes remain poorly understood. In particular, nsLTPs have not been investigated in this genus despite their recognized roles in lipid binding, transport, and stress responses in other plant lineages. While nsLTPs are well characterized in model species, their diversity, structural features, and evolutionary history in non-model taxa such as *Cereus* and *Cipocereus* remain largely unexplored. Here, we characterize the nsLTP repertoire of *Cereus* through an integrative framework combining comparative transcriptomics, phylogenetic reconstruction, structural prediction, binding-site characterization, and codon-based evolutionary analyses. Specifically, we investigate whether sequence variation is preferentially concentrated in regions surrounding predicted ligand-binding cavities while the overall nsLTP structural scaffold remains conserved. By integrating structural and evolutionary information, this study provides a foundation for generating testable hypotheses regarding the diversification of nsLTPs in *Cereus* and their potential functional implications.

## Materials and Methods

### Data Collection and Transcriptome Assembly

Transcriptomic datasets representing seven taxa of *Cereus* and *Cipocereus* were obtained from previous studies of our group (Amaral et al. 2025a; 2025b). RNA sequencing was performed from both root and epidermis, and transcriptomes were assembled and annotated as previously described. Only the predicted nsLTP coding sequences identified from these transcriptomes were used in the present study. Detailed information on sequencing, assembly, quality control, and annotation is available in the original publications. Redundant contigs were clustered with CD-HIT v4.8.1 (Li and Godzik 2006) at a 90% identity threshold. Protein-coding sequences were inferred and used for orthology assignment in OrthoFinder v2.4.0 (Emms and Kelly 2019), generating the orthogroup matrix employed in the present analyses. The nsLTP orthogroup contained 55 protein sequences, all of which originated from transcriptomic assemblies and were used in the subsequent structural and evolutionary analyses (Table S1). The orthogroups were annotated against the nsLTP identified for *C. jamacaru* (Cardoso et al. 2026). The isolated orthogroups with the nsLTP function were used for downstream analysis.

### Sequence Collection and Structural Modeling

Structural predictions of *Cereus* nsLTP homologs were generated by AlphaFold3 (Jumper et al. 2021) using default settings in both cases. The standard AlphaFold confidence output pLDDT (per-residue confidence) was recorded to guide downstream interpretation. Although these approaches yield static structural models, they are known to capture evolutionarily conserved and functionally relevant conformational states, particularly for small, disulfide-stabilized proteins such as nsLTPs. For cross-target comparability, we used the full polypeptide as predicted; no manual trimming or domain excision was applied before alignment.

For structural comparisons, we designated the nsLTP identified in the transcriptome of *C. jamacaru* as the reference scaffold. Structural models were generated for all nsLTP homologs recovered from the transcriptomic datasets included in this study. The *C. jamacaru* sequence was used exclusively as a common reference for pairwise structural comparisons and graphical representation because it represents the best-characterized species of the genus and is widely recognized as the iconic mandacaru cactus. The choice of a single reference ensures consistent interpretation of TM-scores and RMSDs across the dataset while avoiding ambiguity introduced by all-vs-all normalization. We performed structure-only alignments using TM-align. For each mobile model vs the reference, TM-align was executed with the -o flag to write superposed coordinates. TM-align returns three key metrics that we recorded for every pair: (i) TM-score (dimensionless, 0–1), (ii) RMSD (Å) over the aligned Cα subset; and (iii) aligned length. We accepted alignments when TM-align successfully returned all three metrics and produced a superposed PDB, and then, they were inspected in PyMOL. Additionally, structural stereochemical quality was evaluated with PROCHECK (Laskowski et al. 1993), using the standard Ramachandran plot assessment and geometry checks to ensure that modeled structures met expected quality thresholds. Because AlphaFold3 generates a single prediction for each input sequence rather than multiple alternative models, no model selection procedure was required. Instead, structural reliability was assessed using complementary confidence and validation metrics, including pLDDT scores, PROCHECK stereochemical analysis, and comparison with experimentally resolved plant nsLTP structures.

### Mapping and Characterization of Binding Regions

Identification of potential ligand-binding sites was performed using FTSite (Ngan et al. 2012; https://ftsite.bu.edu/) and FTMap (Jones et al. 2022; Kozakov et al. 2015; Ngan et al. 2012; Brenke et al. 2009; https://ftmap.bu.edu/) webservers. We used FTSite and FTMap to identify ligand-binding regions on protein surfaces. Both methods probe the surface with small organic fragments and define consensus binding sites where multiple probe clusters overlap. FTMap further characterizes these clusters as energetically favorable “hot spots.” We used the default FTMap probe set and server settings (see Table S2). Hot-spots identified by FTMap were ranked by cluster strength and mapped onto predicted cavities. Across species and paralogs, high-density probe clusters were frequently observed in regions corresponding to large and highly ligandable pockets, showing qualitative agreement between independent surface-mapping and cavity-detection approaches. This convergence provides independent support for the identification of these predicted binding regions (Table S3).

The FPocket web server (Kochnev and Durrant 2022; https://durrantlab.pitt.edu/fpocketweb/) was employed to characterize the protein further using default parameters (minimum alpha-spheres = 35; minimum cavity volume = 50 Å^3^), and pockets were ranked by their ligandability score, which was subsequently interpreted in the context of compatibility with lipid-like ligands relevant to plant physiology. Only pockets with ligandability score > 0.05 or volume > 200 Å^3^ were retained for comparative discussion. This tool identifies and characterizes protein cavities based on geometric and physicochemical descriptors, including pocket volume, hydrophobicity, and ligandability. Pocket scores, cavity volumes, hydrophobicity estimates, and probe-cluster distributions were interpreted descriptively and were not subjected to formal statistical hypothesis testing.

For comparative analysis of 55 nsLTP structures from different *Cereus* species, FTMap was employed to predict putative ligand-binding regions and evaluate the distribution of surface hot spots among homologous proteins (Table S3). FTMap identifies potential interaction regions by probing protein surfaces with small organic fragments and detecting consensus clusters with favorable binding properties. The main predicted cavities were subsequently characterized using the DoGSite3 module from the ProteinPlus platform (https://proteins.plus/), which evaluates pocket properties based on geometric and physicochemical descriptors.

### Molecular Docking Analyses

To evaluate the predicted ligand-binding properties of the nsLTP structures, molecular docking analyses were performed using AutoDock Vina v1.2.5 (Eberhardt et al. 2021). Representative AlphaFold3 models were used as receptors, while five naturally occurring fatty acids commonly associated with plant lipid metabolism were selected as ligands: lauric acid (C12:0), myristic acid (C14:0), palmitic acid (C16:0), oleic acid (C18:1), and linoleic acid (C18:2). Ligand structures were obtained from the PubChem database and converted to the PDBQT format using Open Babel v3.1.1 (O'Boyle et al. 2011). Protein structures were likewise converted to PDBQT format after the addition of polar hydrogens and assignment of Gasteiger charges. Docking calculations were centered on the ligand-binding pocket predicted by FPocket, using identical search parameters for all receptors and ligands.

Each protein–ligand combination was docked in triplicate, and the best binding affinity (kcal mol⁻^1^) obtained in each run was retained for subsequent analyses. Mean binding energies and standard deviations were calculated for each protein–ligand pair. Statistical comparisons among ligands and among protein models were performed using appropriate statistical tests according to data distribution, with significance assessed at p < 0.05.

### Species Tree, Phylogenetic Reconstruction, and Evolutionary Analysis

The species phylogeny required for gene tree reconciliation analyses were performed using a genome-scale species tree using 719 single-copy orthogroups identified with OrthoFinder. Amino acid sequences from each orthogroup were individually aligned using MAFFT v7.520 (Katoh and Standley 2013) and subsequently analyzed under the multispecies coalescent framework implemented in ASTRAL-III (Zhang et al. 2018), which estimates species relationships from multiple gene trees while accounting for incomplete lineage sorting. The resulting species tree was used as the reference topology for gene tree reconciliation in Notung v2.9 (Stolzer et al. 2012), allowing inference of gene duplication and loss events across the evolutionary history of the genus.

Protein sequences from *Cereus* spp. were aligned using MAFFT v7.520 with the L-INS-i strategy, which provides high accuracy for sequences with conserved motifs and variable loop regions. The resulting alignment was visually inspected in Bioedit 5.0.9 (Hall et al. 2011) to remove ambiguously aligned positions and terminal gaps. Phylogenetic inference using the aligned aminoacids sequences was performed with IQ-TREE2 v2.3.5 (Minh et al. 2020). The best-fit amino acid substitution model (Dayhoff+G4) was selected using ModelFinder implemented in IQ-TREE2. Branch support was assessed with 1,000 ultrafast bootstrap replicates and 1,000 SH-aLRT replicates. Gene duplication events were inferred by reconciling the inferred gene tree with a species tree using Notung v2.9 (Stolzer et al. 2012), allowing identification and visualization of duplication and loss events within the phylogeny (Fig. S1).

To evaluate the conservation of the canonical eight-cysteine motif (8CM), representative nsLTP sequences from experimentally characterized plant species (*Oryza sativa*, *Triticum aestivum*, *Nicotiana tabacum* and *Solanum melongena*) were included in the alignment. Sequence conservation was visualized using sequence logos generated with the *ggseqlogo* package in R (Wagih 2017). Conserved cysteine positions were identified from the multiple sequence alignment and mapped relative to the canonical 8CM motif.

### Individual Sites Subject to Episodic Diversifying Selection

The coding sequences (CDS) of nsLTPs were retrieved from the published genome of *Cereus* (Amaral et al. 2025a). To detect signatures of adaptive evolution, the Mixed Effects Model of Evolution (MEME) was applied through the Datamonkey web server (Weaver et al. 2018; https://www.datamonkey.org), which implements the MEME (Murrell et al 2012) method of the HyPhy software package. MEME estimates the ratio of nonsynonymous to synonymous substitution rates (ω = dN/dS), allowing ω to vary both across sites (fixed effects) and across branches at a given site (random effects). This approach enables the identification of episodic positive selection, meaning adaptive events that occur only on a subset of branches at specific codon positions. Because such variation may reflect adaptive, compensatory, or selectively neutral processes, MEME results were interpreted in conjunction with structural context. The analysis was conducted using a significance threshold of p < 0.05. Codon sites with MEME likelihood ratio test (LRT) *p*-values below this threshold were considered candidates for episodic diversifying selection. Because recombination was not explicitly modeled in the present study, the inferred sites of episodic diversifying selection should be interpreted as candidate sites for future investigation rather than definitive evidence of adaptive evolution.

## Results

### Conservation of the Canonical Eight-Cysteine Motif

**Fig. 1:**
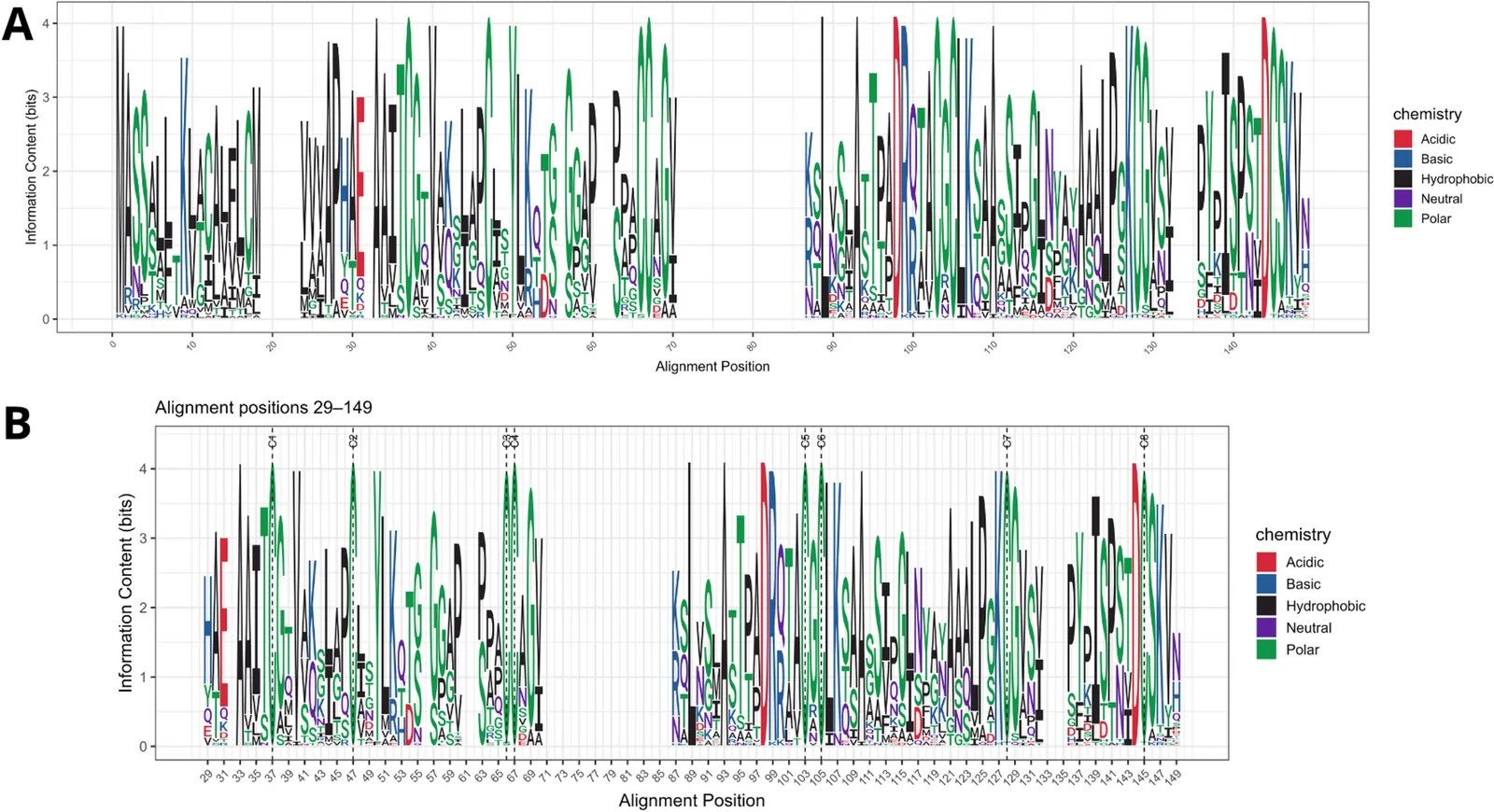
Conservation of the canonical eight-cysteine motif (8CM) in *Cereus* nsLTPs. **A** Sequence logo generated from the multiple sequence alignment of nsLTP proteins. Conserved cysteine residues show the highest information content across the alignment. **B** Enlarged view of the 8CM region showing the eight conserved cysteines (C1-C8) characteristic of plant nsLTPs

A total of 55 nsLTP proteins were recovered from transcriptomic datasets representing seven taxa of *Cereus* and *Cipocereus* (Table S1). Multiple sequence alignment of *Cereus* nsLTPs revealed strong conservation of the canonical eight-cysteine motif (8CM), a defining structural feature of plant nsLTPs (Fig. 1A). All eight cysteine residues were conserved across the analyzed sequences, despite substantial variation in the intervening regions (Table S4). Sequence logo analysis further demonstrated that the highest information content corresponded to the conserved cysteine positions (Fig. 1B). In contrast, residues located between cysteines displayed greater variability, particularly in regions corresponding to loop segments and solvent-exposed surfaces.

This pattern supports the existence of a highly conserved structural scaffold maintained by disulfide-bond networks, while sequence diversification is concentrated in peripheral regions potentially associated with functional fine-tuning. The observed architecture is consistent with the canonical organization described for plant nsLTPs from both monocotyledonous and eudicotyledonous species.

### Structural Assessment of nsLTP Across *Cereus* Species

Structural analyses were performed for all nsLTP homologs identified across the analyzed *Cereus* transcriptomes. To facilitate visualization and avoid excessive redundancy in figure presentation, detailed structural illustrations are shown for the *C. jamacaru* nsLTP reference protein, whereas quantitative structural comparisons were performed across the complete set of homologs.

TM-scores consistently fell within the range typically interpreted as “same topology” (≈0.5–0.7), with aligned cores of ~60–80 residues and backbone RMSDs around 2–3 Å. Importantly, models displaying higher loop divergence also tended to exhibit increased pocket-volume variability and altered rim polarity (Table S2), suggesting a possible association between peripheral structural variation and tunnel geometry.

Across all predicted nsLTP models, mean pLDDT values ranged from 79.22 to 88.97, with an overall average of 84.12 (Table S5). Maximum pLDDT values were consistently high (> 95), indicating strong confidence in the predicted core structures. Residues with pLDDT values below 70 represented, on average, 19.56% of each model, whereas residues below 50 accounted for only 9.55% of the sequence length. These low-confidence regions were predominantly located in solvent-exposed loops and terminal segments (Table S6), while the α-helical scaffold and residues composing the canonical eight-cysteine motif (8CM) generally displayed high confidence scores (Fig. 1). Together with the stereochemical evaluation provided by PROCHECK and the structural agreement with experimentally resolved plant nsLTPs, these results support the reliability of the AlphaFold3 models for downstream comparative analyses.

When the superposed models are inspected, the largest deviations concentrate in solvent-exposed loops and turns that rim the putative binding groove, while the inner β/α framework that shapes the pocket is comparatively conserved. This pattern indicates that static models consistently resolve a structurally constrained core that defines tunnel geometry, while such changes are precisely the ones that modulate ligand accessibility. In practical terms, the *Cereus* nsLTP likely preserves the overall pocket architecture, but varies the local geometry and side-chain presentation at the entrance (loop lengths, insertions/deletions, and substitutions; Table S2). Such changes are precisely the ones that modulate ligand accessibility and local pocket geometry without compromising fold stability (Kader 1996; Yeats and Rose 2008).

**Fig. 2:**
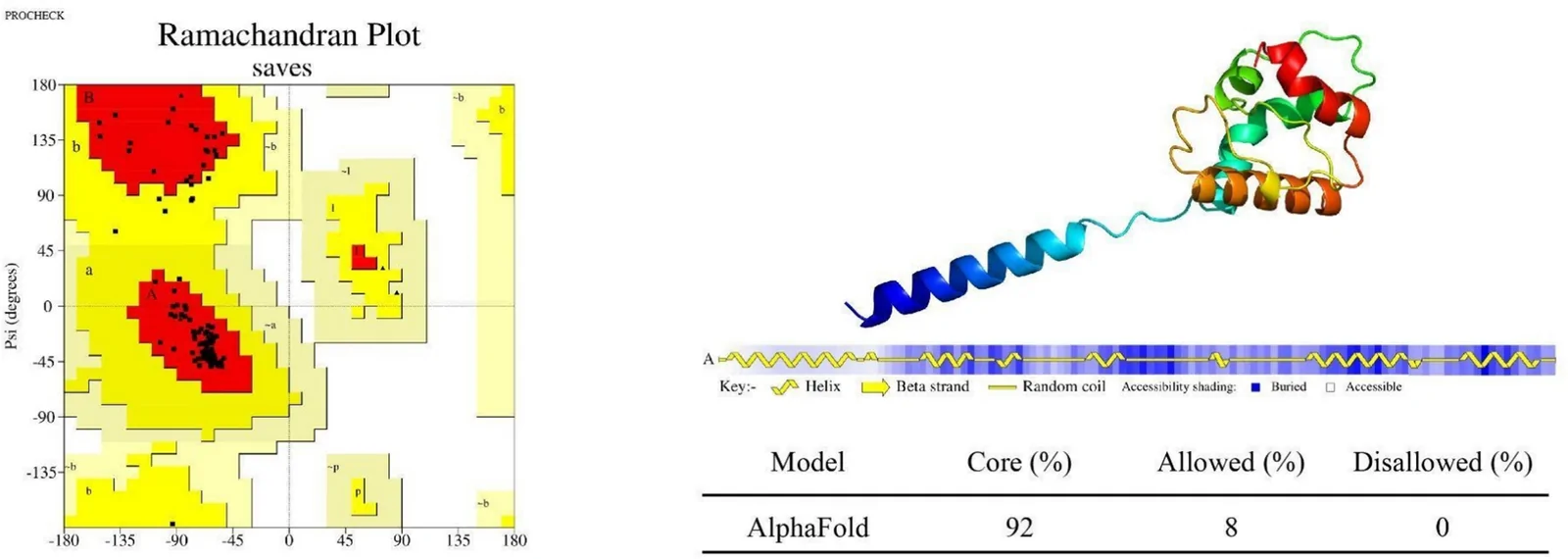
Protein structural quality based on Ramachandran plot. The structure indicates high reliability with most residues in favored regions and none in disallowed regions

The stereochemical analysis of the nsLTP from *Cereus jamacaru* model using PROCHECK shows good structural quality (Fig. 2A, B), with most residues located in favored regions of the Ramachandran plot (92.3%). However, nsLTP (Fig. 2A) shows no bad contacts and slightly smaller deviations in bond lengths and angles. This suggests a higher predicted geometric stability, although these differences must be interpreted cautiously, as static predictions do not capture conformational flexibility. The model has only three residues flagged in the Ramachandran plots, satisfactory side-chain parameters, and exhibits a positive overall G-factors (0.23), suggesting a more reliable global conformation. Thus, while the model is suitable for structural and docking analyses, nsLTP presents marginally better geometric scores. Still, these differences are within the expected range for in silico models, and do not guarantee functional superiority without experimental validation.

The AlphaFold3 model revealed the canonical nsLTP α-helical scaffold with a conserved hydrophobic cavity, consistent with previous structural descriptions of plant nsLTPs. To further place the *Cereus* model into a broader structural context, we compared the predicted structure of *C. jamacaru* with experimentally determined plant nsLTPs available in the Protein Data Bank (Fig. S2). Structural superposition with PDB entries 7W9A (*Solanum melongena*) and 1GH1 (*Triticum aestivum*) confirmed that the overall nsLTP fold is highly conserved despite sequence divergence. The comparison with 7W9A showed very high structural similarity (TM-score = 0.928, TM-align RMSD = 1.16 Å; PyMOL RMSD = 0.624 Å), whereas 1GH1 displayed lower but still significant similarity (TM-score = 0.757, TM-align RMSD = 2.13 Å; PyMOL RMSD = 1.929 Å). These observations reinforce that the structural variation identified in *Cereus* is primarily localized to peripheral regions surrounding the ligand entrance rather than involving major alterations of the conserved protein scaffold. Consequently, our results should not be interpreted as indicating a structural architecture unique to *Cereus*, but instead as evidence that sequence diversification in the proteins analyzed here occurs within a fold broadly conserved among plant nsLTPs.

### Identification of Pockets and Functional Regions

#### Identification and Characterization of Binding Sites

**Fig. 3:**
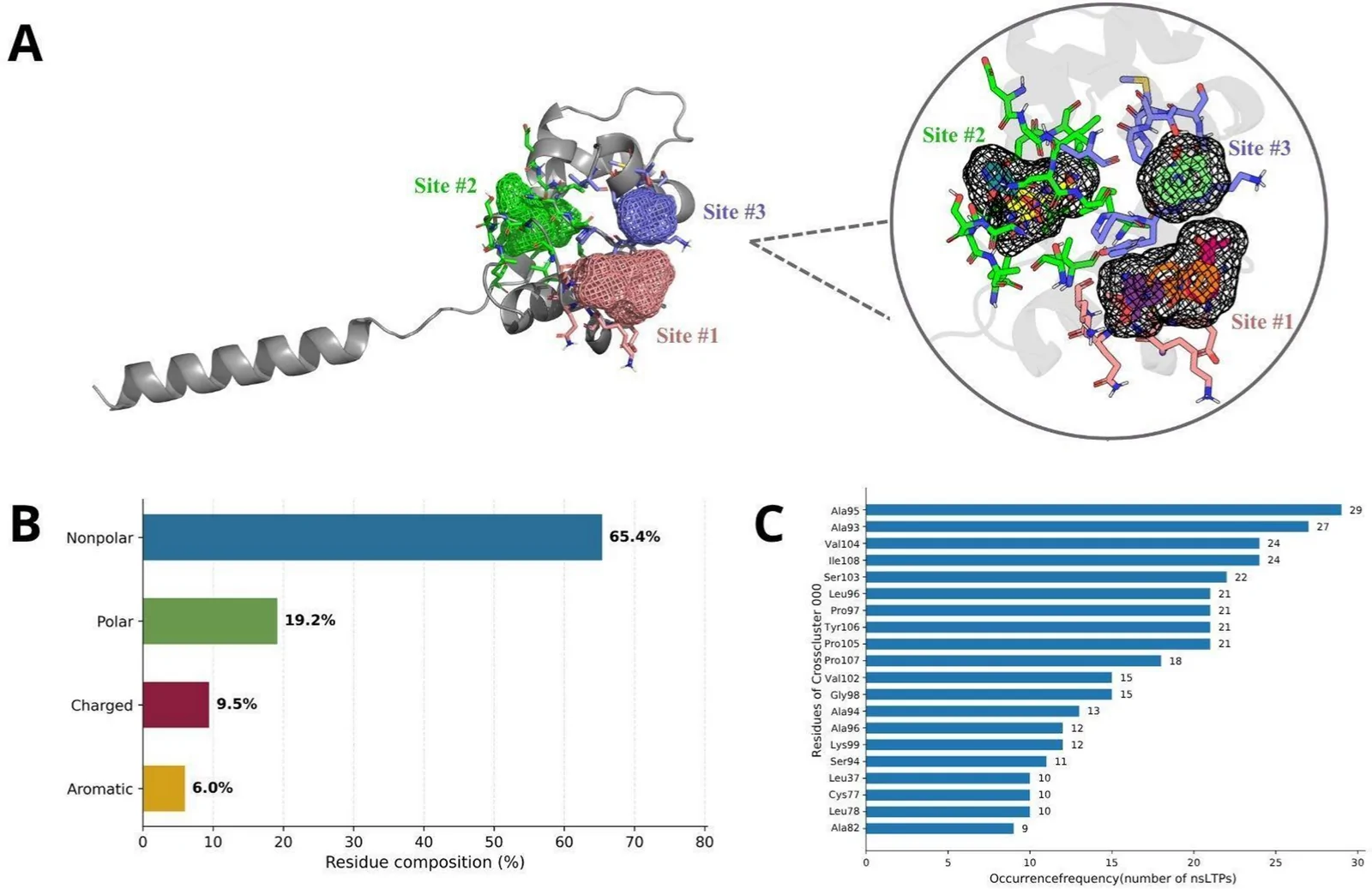
Binding sites and probe clusters in nsLTP structures. **A** Binding site organization and probe clusters identified by FTMap in the nsLTP_AF3 structure. **B** Chemical composition of Crosscluster 000 binding sites by FTMap. **C** Residue conservation in the Crosscluster 000 binding site of FTMap in all 55 structures of genus Cereus

The structure of the nsLTP (Fig. 3 and Fig. S3) was submitted to the online server FTMap to locate potential binding sites and determine its main characteristics. Additionally, both structures were analyzed using FPocket to identify and quantify pockets based on geometric and physicochemical properties, providing complementary information on pocket volume, depth, hydrophobicity, and ligandability. Hydrogen bond interactions for individual residues were further assessed using FTMap, offering detailed insight into residue participation within the predicted binding sites.

The AlphaFold3-predicted structure (Fig. 3) revealed three major binding regions characterized by multiple overlapping FTMap probe clusters (Table 1). Site #1 showed the highest probe diversity, with sixteen probe types distributed across three overlapping clusters, indicating a structurally accessible binding environment consistent with the potential accommodation of ligands with distinct physicochemical properties. The main interacting residues include both hydrophobic (Val75, Pro63) and polar or charged amino acids (Lys65, Asp71, Gln76), suggesting a mixed physicochemical environment compatible with interactions involving amphipathic molecules (Fig. 3B and C).

**Table 1:** Summary of Binding Sites and FTMap Probe Clusters

Binding site	Residues involved	FTMap probe clusters	
		Clusters	Probe
Site #1	Pro63, Tyr64, Lys65, Thr70, Asp71, Cys72, Lys74, Val75, Gln76, Lys77	Cluster 1	CHX, ACD, ACT, BDY, BEN, BUT, ETH, ACN, DME, URE, ADY, DFO, EOL, PHN, THS, AMN
		Cluster 2	BUT, BDY, CHX, BEN, ETH, THS, ACT, ADY, DFO, DME, PHN
		Cluster 3	BUT, ACT, BDY
Site #2	Ile28, Thr32, Val33, Ser36, Leu37, Asp58, Gly59, Ile60, Asn61, Ile62, Ser78, Ser80, Ser81, Thr82, Ala83, Val92, Leu96	Cluster 1	ACD, ACN, ACT, ADY, AMN, BEN, DFO, DME, EOL, ETH, PHN, THS, URE
		Cluster 2	URE, DFO, PHN, ACN
Site #3	Pro63, Tyr64, Lys65, Gly112, Ser113, Met114, Pro115, Thr116, Val120	Single cluster	BDY, CHX, ACD, ACT, ADY, BEN, DFO, ETH, PHN, THS, DME, BUT, EOL, URE, ACN

Sites #2 and #3 also displayed multiple interacting residues and overlapping probe distributions, reinforcing the presence of structurally defined regions with potential ligand-binding capacity.The identified binding sites preserve the characteristic hydrophobic tunnel architecture described for plant nsLTPs (Kader 1996; Yeats and Rose 2008), while exhibiting local variations in residue composition and surface properties that may influence ligand accessibility and binding specificity. The predominance of hydrophobic residues within the cavity is consistent with the established role of nsLTPs in lipid binding and transport, whereas the presence of polar and charged residues near pocket entrances may contribute to ligand recognition and stabilization.

The physicochemical composition of the Crosscluster 000 binding site, as determined by FTMap, further supports the hydrophobic character of the *Cereus* nsLTP cavity (Fig. 3B). Nonpolar residues accounted for the majority of the pocket lining (65.4%), followed by polar (19.2%), charged (9.5%), and aromatic residues (6.0%). This predominance of nonpolar residues is consistent with the lipid-binding function typically attributed to nsLTPs, in which an extensive hydrophobic tunnel accommodates the acyl chains of lipid ligands, while the smaller fractions of polar and charged residues likely contribute to entrance-region interactions and ligand orientation.

Residue-level conservation analysis across the 55 structures of the genus Cereus (Fig. 3C) revealed a consistent pattern of hydrophobic and small-residue conservation within the Crosscluster 000 binding site. Ala95 (29/55) and Ala93 (27/55) were the most frequently observed residues, followed by Val104 and Ile108 (24/55 each). The high conservation of alanine, valine, leucine, and proline residues reinforces the structural stability of the hydrophobic core across species within the genus, while the recurrence of Leu37 and Cys77, also identified among the residues lining Site #2 and Site #1, respectively (Table 1) suggests that these positions may play a conserved functional role in ligand accommodation, independent of the specific binding subsite considered.

#### Structural Pocket Analysis

Pocket analysis of the nsLTP structure identified five potential binding cavities with distinct physicochemical properties (Fig. 3A). Among these, pocket #1 exhibited the highest pocket score (0.368) and the largest volume (452.3 Å^3^), indicating a geometrically favorable and accessible region for ligand accommodation. This cavity also displayed a balanced combination of apolar and polar surface areas, suggesting compatibility with lipid-like and amphipathic molecules. Pockets #2 and #5 were predominantly hydrophobic (apolar SASA > 84%), consistent with potential interactions involving lipid or hydrocarbon ligands. In contrast, pockets #3 and #4 exhibited higher polarity and intermediate volumes (259–391 Å^3^), indicating the possibility of accommodating molecules with mixed physicochemical characteristics. The predominance of hydrophobic surface area across most cavities is consistent with the established role of nsLTPs in lipid binding and transport. Among the identified sites, pocket #1 represents the most promising candidate for ligand interaction due to its combination of volume, accessibility, and ligandability score. Together, these results support the presence of a structurally defined binding region compatible with the canonical lipid-binding function of plant nsLTPs.

Previous work by our group (Cardoso et al. 2026) has demonstrated the feasibility of combining large-scale molecular docking with molecular dynamics simulations using triacylglycerols and related lipid substrates in *Cereus* proteins. These analyses highlighted both the potential and the methodological challenges associated with modeling long-chain and highly flexible lipid molecules in dynamic environments.

#### Molecular Docking Supports Ligand-Dependent Differences Among nsLTP Variants

The representative nsLTP models were subjected to molecular docking against a panel of lipid-related ligands to investigate whether the structural variation observed around the predicted binding pockets could influence ligand recognition. Overall, docking scores differed significantly among ligands and protein variants (Table S6), indicating that the predicted binding cavities are not energetically equivalent across the analyzed nsLTPs. While all proteins retained the characteristic hydrophobic tunnel of plant nsLTPs, the magnitude of the predicted binding energies varied according to both ligand identity and local pocket geometry.

The strongest predicted interactions were consistently associated with ligands compatible with the hydrophobic cavity described for plant nsLTPs (Kader 1996; Madni et al. 2020), whereas other ligands exhibited weaker or more variable docking scores among homologs. These differences are consistent with the structural variability observed in solvent-exposed loops surrounding the tunnel entrance, suggesting that peripheral structural diversification may influence ligand recognition without disrupting the conserved nsLTP scaffold. Because molecular docking evaluates static binding compatibility rather than lipid transfer or binding kinetics, these results should be interpreted as computational evidence supporting the proposed gate-modulation framework rather than direct evidence of altered biological function.

### Phylogeny and Evolutionary Context of the Gene/Protein

The species tree inferred from 719 single-copy orthologs recovered a well-resolved phylogenetic framework for the sampled taxa (Fig. S1). *C. calcirupicola* occupied the earliest-diverging position within the sampled Cereus species, whereas the remaining taxa formed a second major lineage. Within this lineage, *C. jamacaru* diverged prior to the clade containing *C. pierre-braunianus, C. fernambucensis, C. mirabella, C. hildmannianus*, and the outgroup *Cipocereus bradei*. This species phylogeny was subsequently used as the reference topology for reconciliation analyses of the nsLTP gene family.

**Fig. 4:**
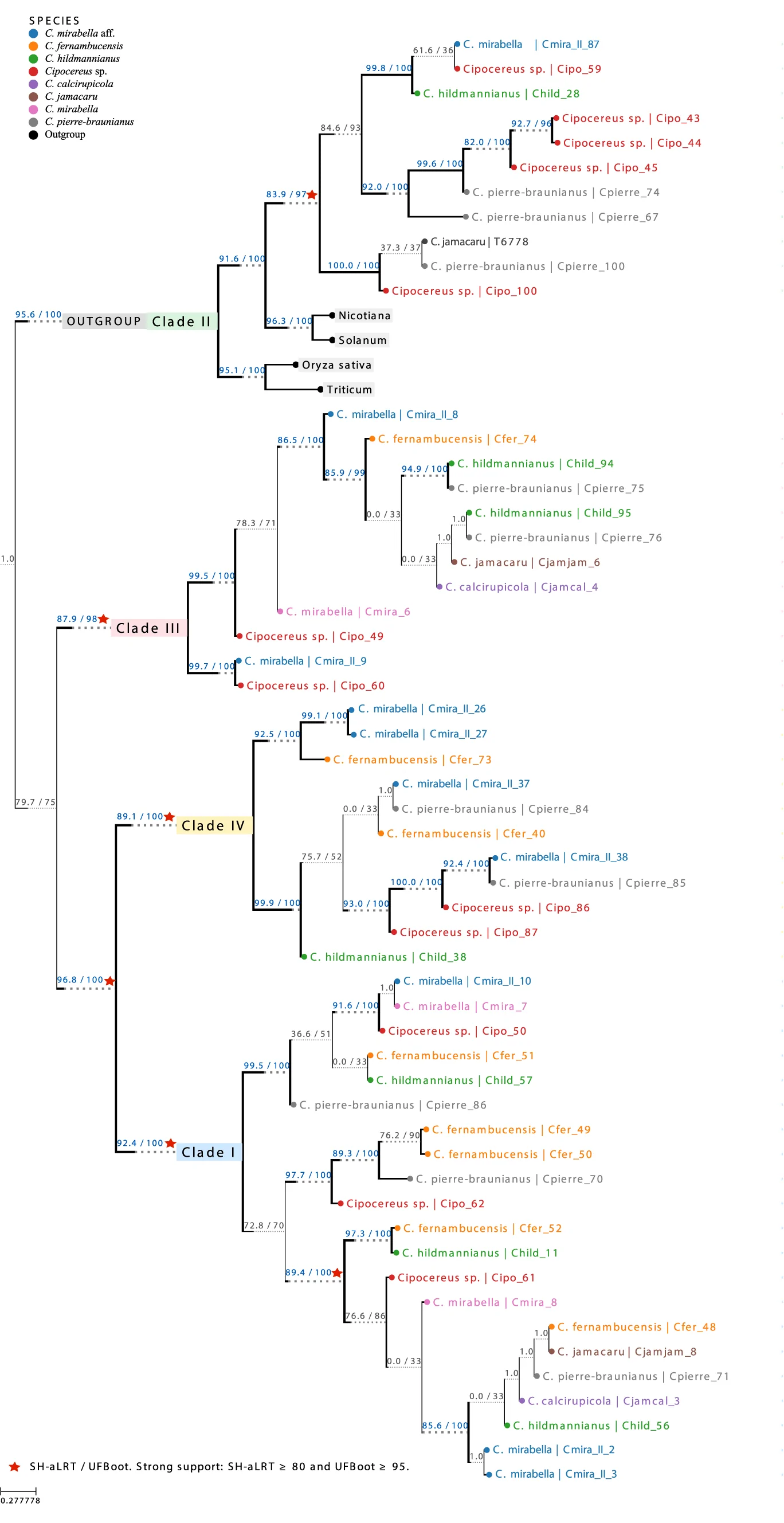
Maximum-likelihood phylogeny of *Cereus* nsLTPs inferred with IQ-TREE2 under the Dayhoff+G4 model. Branch support values are shown as SH-aLRT/UFBoot. Terminal labels indicate species and gene/transcript identifiers, with colors representing species of origin. Red stars denote gene duplication events inferred by reconciliation with the genome-scale species tree. Four major *Cereus* clades (I–IV) were identified. Representative nsLTPs from *Nicotiana tabacum*, *Solanum melongena*, *Oryza sativa*, and *Triticum aestivum* were included as outgroups

Maximum-likelihood phylogenetic analysis recovered all *Cereus* nsLTPs within a single, well-supported lineage relative to the representative plant nsLTPs included as external references (Fig. 4), supporting the monophyletic origin of this gene family within the genus. The phylogeny was inferred under the Dayhoff+G4 substitution model selected by ModelFinder, and branch support was assessed using both SH-aLRT and ultrafast bootstrap analyses. Several terminal clades exhibited strong statistical support (SH-aLRT ≥ 80; UFBoot ≥ 95), whereas a limited number of deeper relationships showed lower support and were therefore interpreted cautiously.

Orthologous nsLTP sequences from different *Cereus* species were extensively intermingled throughout the phylogeny rather than forming species-specific clusters. This pattern indicates that a substantial proportion of the diversification of the family predates the radiation of extant *Cereus* species and that many duplicated copies have been retained across descendant lineages. At the same time, several terminal clades contained multiple copies from the same species, revealing lineage-specific paralogous duplications superimposed on this ancestral diversification pattern. Thus, the evolutionary history of the family appears to involve both ancient duplication events shared among species and more recent duplication events restricted to individual evolutionary lineages.

To provide an explicit evolutionary framework for reconciliation analyses, a genome-scale species tree was independently inferred from 719 single-copy orthogroups identified by OrthoFinder and analyzed under the multispecies coalescent using ASTRAL-III (Fig. S1). This species phylogeny was subsequently employed for reconciliation analyses in Notung, allowing inference of gene duplication and loss events while accounting for the evolutionary relationships among species. Because reconciliation depends on the underlying gene-tree topology, biological interpretations of duplication and loss events were restricted primarily to well-supported regions of the phylogeny, whereas deeper nodes with lower statistical support were interpreted conservatively.

The coexistence of broadly distributed orthologous groups and lineage-specific paralogs suggests that the expansion of the *Cereus* nsLTP family follows the birth-and-death model commonly described for multigene families in plants (Lynch and Conery 2000; Edstam et al. 2011). Under this evolutionary model, duplicated genes may be retained, lost, or gradually diverge following duplication, generating heterogeneous gene repertoires among closely related species while preserving the conserved molecular framework characteristic of the family. The widespread retention of orthologous copies across species indicates that much of the ancestral nsLTP repertoire has remained evolutionarily stable, whereas lineage-specific paralogs probably reflect more recent evolutionary events contributing to local diversification. Because some internal relationships showed limited statistical support, duplication and loss events associated with poorly supported nodes were interpreted with caution.

### Identification of Coding Sites Under Positive Selection Across the Phylogeny

**Fig. 5:**
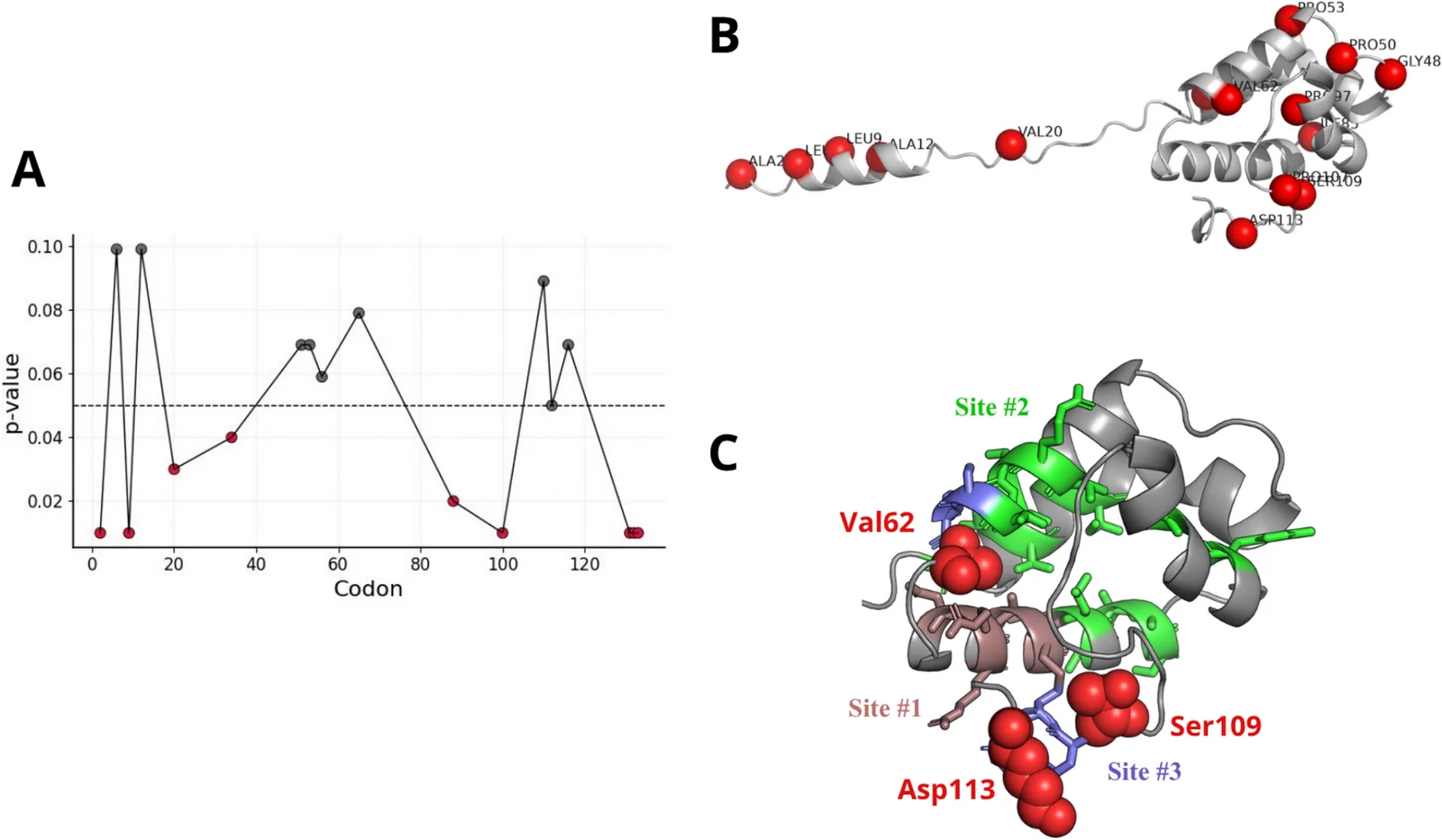
Evidence of positive selection and structural–functional mapping of the analyzed protein*.* **A** Codon sites under positive selection detected by the MEME (Mixed Effects Model of Evolution) analysis. Each point represents a codon with a statistically significant signal of diversifying selection (*p*-value). **B** Predicted 3D structure generated by AlphaFold from individual ID Cjamjam_8 as an example. Codon positions under positive selection are highlighted as orange spheres. **C** Structural overlap between positively selected residues (red spheres) and three predicted functional binding sites: *Site 1* (light red), *Site 2* (green), and *Site 3* (violet). Several positively selected residues are spatially adjacent to functional regions, suggesting potential adaptive relevance for binding or molecular interaction interfaces

The analysis using MEME (Mixed Effects Model of Evolution) on Datamonkey was conducted with 39 aligned sequences, totaling 133 codon sites. Of the 133 sites evaluated, 18 showed evidence of episodic positive selection (Fig. 5), corresponding to about 13.5% of the total. From them, 9 sites displayed *p* < 0.05 (Table S8); the others did not reach *p* < 0.05. On average, each codon under positive selection was detected in 1 to 2 branches of the phylogenetic tree (Table S8), suggesting that selective pressure does not act uniformly across the entire gene but rather locally in specific clades or lineages. This pattern is consistent with possible episodic functional fine-tuning, although alternative explanations, including compensatory evolution, lineage-specific constraint relaxation, or the influence of historical recombination, cannot be excluded. Consequently, the MEME results should be interpreted as identifying candidate codons associated with episodic sequence diversification rather than definitive targets of adaptive evolution. This result is consistent with lineage-specific diversification during the evolutionary history of the group, although the functional consequences of these substitutions remain unknown (Fig. 5C). The fact that no sites were identified with variable ω (dN/dS) across all branches reinforces (Table S8) the idea that the observed positive selection is episodic and restricted rather than pervasive adaptive pressure.

Mapping of candidate codons detected by MEME onto the structural models showed that several of these residues are located in solvent-exposed loops or near residues surrounding the predicted tunnel entrance. These observations provide structural context for the MEME results, but they should not be interpreted as evidence of statistically significant spatial enrichment. Because no formal permutation or enrichment test was performed, the association between candidate sites and pocket-adjacent regions is discussed only as a qualitative pattern and as a hypothesis for future functional validation.

## Discussion

Our analyses support a proposed gate-modulation framework for *Cereus* nsLTPs, in which a conserved four-helix scaffold maintains the hydrophobic tunnel, while loop-level diversification at rim/entrance regions tunes tunnel accessibility and local physicochemical properties. By integrating structural comparisons, pocket/hot-spot mapping, phylogenetic context, and episodic selection signals, we show that functional tunability is concentrated in peripheral, flexible regions rather than in the structural core. This provides a mechanistic bridge between molecular variation and physiological roles commonly attributed to nsLTPs (e.g., lipid trafficking linked to cuticle/membrane function under stress), without implying direct ecological causality in the absence of functional assays. Because several candidate codons occur within structurally flexible loop regions, local alignment uncertainty cannot be completely excluded. Nevertheless, the conservation of the canonical eight-cysteine motif (8CM), together with the high-confidence AlphaFold3 predictions, supports the overall reliability of the sequence alignment and the inferred structural framework. Therefore, the MEME results should be interpreted as candidate sites for future functional investigation rather than definitive evidence of adaptive functional divergence.

### Structure Modelling Quality and Characterization for *Cereus* nsLTP

The AlphaFold3 models revealed a conserved structural organization characteristic of plant nsLTPs, consisting of a compact α-helical scaffold stabilized by the canonical eight-cysteine motif (8CM) and surrounding a central hydrophobic cavity (Salminen et al. 2016). Pocket and cavity scores were interpreted here as structural descriptors of ligandability, providing information on the geometric and physicochemical properties of regions potentially compatible with lipid-like molecules involved in plant physiology.

Three major binding regions were identified by FTMap and FTSite, with Site #1 exhibiting the highest probe density and the largest overlap among fragment clusters (Table 1; Fig. 3). This region contains a combination of hydrophobic residues, including Val75 and Pro63, together with polar and charged residues such as Lys65, Asp71, and Gln76, generating a mixed physicochemical environment potentially compatible with interactions involving amphipathic molecules. Similar arrangements have been described in experimentally characterized plant nsLTPs, where ligand-binding cavities are formed by residues distributed across multiple α-helices and connected loop regions (Simorre et al. 1991; Gincel et al. 1994; Salminen et al. 2016). This predominance of nonpolar residues, with polar and charged residues restricted mainly to the pocket entrance, mirrors the entrance-lid mechanism described for Arg44/Tyr79 in nsLTP1s (Missaoui et al. 2022), reinforcing that ligand accommodation relies primarily on hydrophobic packing rather than specific polar interactions.

Because AlphaFold3 generates static structural predictions, the dynamic behavior of these regions cannot be directly evaluated. Consequently, interpretations regarding conformational flexibility, ligand-induced rearrangements, or alternative structural states require future investigation through molecular dynamics simulations or experimental structural approaches (Krokidis et al. 2025). Nevertheless, the high confidence scores observed for the α-helical scaffold and the consistent identification of binding regions across complementary pocket-detection methods support the structural reliability of the predicted models and their suitability for comparative evolutionary analyses.

The docking analyses further support this interpretation by showing that predicted binding energies vary among nsLTP homologs despite preservation of the conserved hydrophobic scaffold (Table S4). Although docking does not provide direct evidence of lipid transfer or binding specificity, the observed differences are consistent with the hypothesis that structural variation surrounding the pocket entrance contributes to differences in ligand compatibility. Similar relationships between local pocket architecture and ligand recognition have been described for plant nsLTPs and other lipid-binding proteins (Madni et al. 2020; Hamaï and Drin 2024).

The functional implications of these structural differences are particularly relevant in light of the multifunctional nature of nsLTPs. These proteins are known to participate in diverse physiological processes, from cuticle formation and pathogen defense to lipid signaling and membrane remodeling (Shenkarev et al. 2017; Madni et al. 2020). However, it remains unclear whether such functional diversity arises from the existence of multiple isoforms or from the intrinsic ability of nsLTPs to accommodate a broad range of lipid molecules.

Many positively selected codons mapped to solvent-exposed loops surrounding the predicted pocket entrance, suggesting that these regions deserve future functional investigation. However, because MEME does not distinguish among adaptive, compensatory or neutral processes, this association should be regarded as a hypothesis.

### Structural Comparison

Comparative structural analyses performed across all identified *Cereus* nsLTP homologs indicate that these proteins preserve the same global architecture observed across the homologous set (Fleury et al. 2019; Fig. 3). The combination of high topological agreement with modest geometric spread supports the view that the *Cereus* protein is not a new fold among them, but rather a moderately diverged member of the family (Santos-Silva et al. 2023). The residues that fail to align one-to-one are largely confined to peripheral segments, whereas the inner β/α framework that defines the pocket is retained. The concentration of sequence and structural variation in loop regions should not be interpreted as a feature unique to *Cereus*. Similar patterns have been reported for nsLTP families from both monocotyledonous and eudicotyledonous plants. Our results support that this general evolutionary framework is also present within Cereus and provide the first integrated structural and evolutionary characterization of this pattern for the genus.

Despite this fold-level conservation, the largest deviations cluster in solvent-exposed loops and turns that rim the binding groove (Malinina et al. 2017). These insertions/deletions and side-chain substitutions reshape the local geometry and electrostatics at the pocket entrance without disrupting the core cavity. Functionally, such “rim remodeling” is a well-known route to tune specificity and affinity while preserving catalytic or binding competence (Hamaï and Drin 2024). Thus, the most parsimonious interpretation is structural variation around the binding site superimposed on a conserved scaffold (Fig. 3). The observed structural variation may influence ligand recognition properties (e.g., shifts in K_D_, K_M_, or K_cat_ depending on the biochemical role of nsLTP), although this remains to be experimentally tested. The conserved fold provides a common structural framework, whereas variation in peripheral loops may contribute to differences in ligand recognition among homologs. Whether these differences translate into functional specialization remains to be experimentally demonstrated. This interpretation is further supported by the molecular docking analyses, which revealed ligand-dependent differences in predicted binding energies among structurally similar nsLTPs (Table S7). These findings suggest that relatively small structural changes concentrated around tunnel entrances may alter ligand compatibility while preserving the conserved four-helix scaffold, consistent with previous observations that peripheral regions frequently modulate ligand recognition in lipid-binding proteins (Hamaï and Drin 2024; Madni et al. 2020).

Two caveats temper these conclusions. Most observed differences arise in low-confidence, high-flexibility regions by their very nature (loops), and single static models cannot capture their full conformational ensembles (Srinivasan et al. 2024). Nevertheless, the consistent localization of structural variation at tunnel rims across independent modeling approaches suggest that this recurrent pattern is unlikely to result exclusively from isolated modeling artifacts (Hasegawa and Holm 2009). Static structures therefore provide a reliable framework for identifying evolutionarily stable features and functionally tunable interfaces in nsLTPs.

These observations support a model in which *Cereus* nsLTPs preserve the canonical structural scaffold characteristic of the family while accumulating variation in loop regions and tunnel-rim residues. Such variation is expected to influence local physicochemical properties and ligand accessibility without disrupting overall fold stability. The concentration of episodically variable codons in these structurally flexible regions further supports the hypothesis that diversification has occurred primarily through localized modifications rather than large-scale structural innovation.

### Ecological and Adaptive Significance

Previous studies have associated nsLTPs with lipid transport, cuticle formation, wax deposition, membrane remodeling, and responses to environmental stress in several plant species (Deeken et al. 2016; Rojas et al. 2019; Xue et al. 2022; Xiao et al. 2023). In xerophytic and drought-tolerant systems, these proteins have been linked to processes involved in maintaining cellular and surface integrity under challenging environmental conditions. Although the present study does not directly evaluate these functions, the structural and evolutionary patterns observed in *Cereus* are compatible with the broader functional diversity reported for the nsLTP family.

The presence of species-specific paralogs, together with signatures of episodic positive selection concentrated in loop regions and pocket-adjacent surfaces, suggests that diversification within the *Cereus* nsLTP family occurred primarily outside the conserved structural core. Such variation may contribute to differences in ligand-binding properties among homologs while preserving the overall fold characteristic of plant nsLTPs (Kader 1996; Edstam et al. 2011). However, the functional consequences of these substitutions cannot be inferred from structural predictions alone. The availability of an independently inferred genome-scale species tree allowed duplication history to be interpreted within an explicit evolutionary framework rather than solely from the nsLTP gene tree. Although several deep nodes of the gene tree exhibited limited statistical support, reconciliation analyses consistently indicated that the expansion of the nsLTP family involved ancestral duplication events followed by lineage-specific retention and loss. This pattern is consistent with the evolutionary dynamics reported for multigene families in plants, where differential retention of duplicated copies contributes to functional diversification while preserving the ancestral structural scaffold (Lynch and Conery 2000; Panchy et al. 2016).

Therefore, the results presented here should be interpreted as generating hypotheses regarding the evolutionary diversification of nsLTPs in *Cereus* rather than providing direct evidence of ecological adaptation or physiological specialization. Future studies integrating biochemical assays, ligand-binding experiments, molecular dynamics simulations, gene expression analyses, and comparative physiological approaches will be necessary to determine whether the observed molecular differences influence protein function and contribute to stress-related processes previously reported for nsLTPs in other plant lineages (Tapia et al. 2013; Liu et al. 2022; Xiao et al. 2023). An additional aspect emerging from the MEME analysis is that episodic diversifying selection affected only one or a few branches for each candidate codon rather than acting consistently across the entire phylogeny. This pattern suggests that sequence diversification occurred through multiple independent evolutionary events after lineage divergence, rather than through pervasive selection affecting all nsLTP copies. Together with the coexistence of ancestral orthologs and lineage-specific paralogs, these observations are consistent with localized evolutionary diversification within a conserved protein family. Although the biological significance of individual substitutions cannot be determined from sequence analyses alone, these candidate sites provide a rational starting point for future biochemical and functional studies.

### Biotechnological Potential

The identification of pocket #1 in the nsLTP model as a high-ligandability cavity for lipid-like molecules highlights the functional versatility of nsLTPs. Here, pocket scores are interpreted as descriptors of structural compatibility with physiologically relevant hydrophobic ligand. This consolidated cavity reflects a structurally optimized region for lipid binding and transport, consistent with the primary biological roles of plant nsLTPs.

These findings align with previous crystallographic studies showing that surface binding of myristic acid near the N-terminal cavity induces conformational changes in loop-3, facilitating lipid internalization into the hydrophobic cavity, while binding near the C-terminal end induces only minor structural deviations (Lev 2010; Madni et al. 2020; Leighton et al. 2026). These studies also support a model of largely unidirectional lipid transfer, with lipids entering from the N-terminal end, moving through the hydrophobic cavity, and exiting at the C-terminal end, coordinated by loop-3 and C-terminal loop movements. Similar to the unexpected lipolytic activity reported for Cor a 8 in *C. avellana*, which operates without a classical catalytic triad (Fissore et al. 2025), the presence of a structurally compact and accessible pocket indicates that nsLTPs may accommodate diverse ligands and potentially perform catalytic or transport functions that remain underexplored. While such findings expand the functional landscape of nsLTPs, the present study is focused on structural, evolutionary, and ligand-binding features of *Cereus* nsLTPs and does not provide experimental or computational evidence for catalytic activity. Therefore, any potential relationship between *Cereus* nsLTPs and lipolytic functions should be regarded as a working hypothesis, to be addressed in future studies combining biochemical assays and dynamic simulations.

Key residues such as Arg44 and Tyr79 in nsLTP1s (Finkina et al. 2016; Melnikova et al. 2018; Missaoui et al. 2022) participate in ligand binding, with additional residues stabilizing interactions with polar heads and aliphatic chains. Regulatory features, including calmodulin-binding regions and phosphorylation sites, may further modulate lipid uptake, linking nsLTP function to stress responses and plant adaptation (Chiu et al. 2020). These structural insights, combined with high stability and compact folding of nsLTPs, make them attractive scaffolds for in silico docking studies, molecular dynamics simulations, and potential synthetic biology or biomaterial applications.

Functionally, nsLTPs contribute to both biotic and abiotic stress tolerance. Transgenic plants overexpressing nsLTPs demonstrate increased resistance to fungi, bacteria, and oomycetes, and show enhanced tolerance to drought, salinity, cold, and oxidative stress (McLaughlin et al. 2021; Xu et al. 2018). Their ability to act synergistically with other antimicrobial peptides, mediate cutin and wax deposition, and regulate trichome development underlines their versatile roles in plant defense and adaptation (Meng et al. 2018; Song et al. 2020).

Taken together, the structural, mechanistic, and physiological insights into nsLTPs suggest multiple avenues for future research. High-ligandability pockets, particularly in *Cereus* nsLTP, can be explored for ligand docking and bioactive compound screening. The molecular docking analyses performed in this study demonstrate that the predicted binding pockets are compatible with lipid-like ligands and that binding energies differ among nsLTP homologs (Table S7). Although these predictions require experimental validation, they provide computational support for the hypothesis that structural diversification within the *Cereus* nsLTP family may influence ligand recognition. Consequently, these proteins represent promising targets for future biochemical characterization and rational engineering of lipid-binding proteins.

## Conclusion

This study provides the first comprehensive structural and evolutionary characterization of nsLTPs in the genus *Cereus*, revealing structural and evolutionary patterns potentially relevant to adaptation in arid and semi-arid environments. Our integrated approach combining comparative transcriptomics, homology-based modeling, binding-pocket analysis, and evolutionary inference demonstrates that *Cereus* nsLTPs preserve the canonical four-helix hydrophobic-tunnel architecture typical of the family, while exhibiting local flexibility at loop regions surrounding the binding pocket. This conserved-core yet variable-rim pattern supports functional tunability: loop variation and episodic positive selection may act as fine-scale mechanisms to modulate ligand affinity, lipid specificity, and potentially stress-related signaling. Such structural-functional plasticity may influence membrane protection and lipid trafficking under abiotic stress condition, potentially contributing to the ecological resilience and biogeographical breadth of the genus. In addition, molecular docking analyses provided independent computational support for differences in predicted ligand compatibility among nsLTP homologs, reinforcing the proposed gate-modulation hypothesis while highlighting the need for future biochemical and dynamic validation.

Nine codon sites under episodic diversifying selection, together with species-specific paralogs, indicate ongoing lineage-specific diversification rather than radical innovation. These features suggest that nsLTP diversification in *Cereus* followed a model of constrained innovation, where small structural adjustments at the interface level generate functional diversity without compromising scaffold stability. Moreover, the identification of highly ligandable pockets points to potential biotechnological applications, as nsLTPs may serve as molecular scaffolds for lipid-binding, delivery systems, or bioactive compound transport.

## Supplementary Information

Below is the link to the electronic supplementary material.

Supplementary file1 (DOCX 1009 KB)

Supplementary file2 (XLSX 823 KB)

## Data Availability

No datasets were generated or analysed during the current study.
