# Supplementary material for: Structural and Evolutionary Analysis of Non-specific Lipid Transfer Proteins (nsLTPs) in Cereus (Cactaceae)
Source: J Mol Evol. 2026 Oct 9;94(5):1002–18. doi: 10.1007/s00239-026-10351-2 (PMC13672417; doi:10.1007/s00239-026-10351-2)
Supplement: Supplementary file 1 [file 239_2026_10351_MOESM1_ESM.docx]

**Structural and evolutionary analysis of non-specific lipid transfer proteins (nsLTPs) in *Cereus* (Cactaceae)**

Cardoso, M.I.O^1^, Teodoro, J.A.^1^, Costa-Silva, T.A.^1^, Amaral, D.T.^1,*^

^1^Centro de Ciências Naturais e Humanas, Universidade Federal do ABC (UFABC), Santo André, São Paulo, Brazil.

^*^Corresponding author. Avenida dos Estados, 5001, bloco A, Lab. 504-3, Santo André, 09210-580, Brazil. E-mail address: [danilo.trabuco@ufabc.edu.br](mailto:danilo.trabuco@ufabc.edu.br)

**Figure S1.** Genome-scale species tree used for gene tree reconciliation in Notung. The species tree was inferred using ASTRAL-III from 719 single-copy orthologs and was used as the reference topology for inferring gene duplication and loss events in the *Cereus* nsLTP family.

**A**

**B**

**Figure S2.** Structural comparison between the *Cereus jamacaru* nsLTP model and experimentally characterized plant nsLTPs. (A) Crystal structure 7W9A *Solanum melongena* (green; TM-score = 0.928; TM-align RMSD = 1.16 Å). (B) Crystal structure 1GH1 *Triticum aestivum* (blue; TM-score = 0.757; TM-align RMSD = 2.13 Å).

**Figure S3. Hydrogen bond interactions predicted by FTMAP in the protein structures.** (A) Table of H-bond interactions and the structure predicted. Residues forming hydrogen bonds and their interaction frequencies are indicated, highlighting the most probable binding sites.
